# Identification of pathways required for *Salmonella* to colonize alfalfa using TraDIS-*Xpress*

**DOI:** 10.1128/aem.00139-24

**Published:** 2024-06-21

**Authors:** Emma R. Holden, Justin Abi Assaf, Haider Al-Khanaq, Noemie Vimont, Mark A. Webber, Eleftheria Trampari

**Affiliations:** 1 Quadram Institute Bioscience, Norwich Research Park, Norwich, United Kingdom; 2 Norwich Medical School, University of East Anglia, Norwich Research Park, Norwich, United Kingdom; Anses, Maisons-Alfort Laboratory for Food Safety, Maisons-Alfort, France

**Keywords:** TraDIS, foodborne pathogens, fresh produce, *Salmonella*, functional genomics, food safety

## Abstract

Enteropathogenic bacteria, such as *Salmonella*, have been linked to numerous fresh produce outbreaks, posing a significant public health threat. The ability of *Salmonella* to persist on fresh produce for extended periods is partly attributed to its capacity to form biofilms, which pose a challenge to food decontamination and can increase pathogenic bacterial load in the food chain. Preventing *Salmonella* colonization of food products and food processing environments is crucial for reducing the incidence of foodborne outbreaks. Understanding the mechanisms of establishment on fresh produce will inform the development of decontamination approaches. We used Transposon-Directed Insertion site Sequencing (TraDIS-*Xpress*) to investigate the mechanisms used by *Salmonella enterica* serovar Typhimurium to colonize and establish on fresh produce over time. We established an alfalfa colonization model and compared the findings to those obtained from glass surfaces. Our research identified distinct mechanisms required for *Salmonella* establishment on alfalfa compared with glass surfaces over time. These include the type III secretion system (*sirC*), Fe-S cluster assembly (*iscA*), curcumin degradation (*curA*), and copper tolerance (*cueR*). Shared pathways across surfaces included NADH hydrogenase synthesis (*nuoA* and *nuoB),* fimbrial regulation (*fimA* and *fimZ*), stress response (*rpoS),* LPS O-antigen synthesis (*rfbJ),* iron acquisition (*ybaN*), and ethanolamine utilization (*eutT* and *eutQ*). Notably, flagellum biosynthesis differentially impacted the colonization of biotic and abiotic environments over time. Understanding the genetic underpinnings of *Salmonella* establishment on both biotic and abiotic surfaces over time offers valuable insights that can inform the development of targeted antibacterial therapeutics, ultimately enhancing food safety throughout the food processing chain.

**IMPORTANCE:**

*Salmonella* is the second most costly foodborne illness in the United Kingdom, accounting for £0.2 billion annually, with numerous outbreaks linked to fresh produce, such as leafy greens, cucumbers, tomatoes, and alfalfa sprouts. The ability of *Salmonella* to colonize and establish itself in fresh produce poses a significant challenge, hindering decontamination efforts and increasing the risk of illness. Understanding the key mechanisms of *Salmonella* to colonize plants over time is key to finding new ways to prevent and control contamination of fresh produce. This study identified genes and pathways important for *Salmonella* colonization of alfalfa and compared those with colonization of glass using a genome-wide screen. Genes with roles in flagellum biosynthesis, lipopolysaccharide production, and stringent response regulation varied in their significance between plants and glass. This work deepens our understanding of the requirements for plant colonization by *Salmonella*, revealing how gene essentiality changes over time and in different environments. This knowledge is key to developing effective strategies to reduce the risk of foodborne disease.

## INTRODUCTION

Enteropathogenic bacteria present an evolving threat to public health. Historically, these pathogens were predominantly linked to meat products. However, in recent years, fresh produce is emerging as a major cause of these outbreaks, being implicated in over a third of reported outbreaks in certain countries (1). The majority of cases are associated with ready-to-eat crops, although some cases have been attributed to the mishandling of vegetables that are typically subjected to cooking processes (2). Certain human pathogens, such as *Salmonella*, exhibit increased adaptability to colonizing various ecological niches and surviving outside their primary host (3). *Salmonella enterica* has been implicated in numerous recent multistate outbreaks associated with contaminated fruits and vegetables, including lettuce, tomatoes, alfalfa, cucumbers, and melons (4–7). Recent studies have demonstrated that *Salmonella* can actively colonize plant tissues by using specific mechanisms (8). *Salmonella* has been found to persist in produce for extended periods, with viability lasting over 6 months after initial colonization of the pathogen (9).

*Salmonella*’s adaptive strategy to persist in the challenging plant environment includes the formation of biofilms. Biofilms are structured, aggregated communities of microorganisms encased in an extracellular matrix and attached to surfaces (10). These communities play a critical role in enabling pathogenic bacteria to adhere to fresh produce, increasing the risk of enteric disease transmission (11). Bacteria within biofilms exhibit intrinsic tolerance to high concentrations of antimicrobials, biocides, and disinfectants, which complicates decontamination efforts and poses challenges for ensuring food safety (12). Previous studies have contributed valuable insights into the mechanisms underlying the biofilm formation of *Salmonella* and its ability to persist on plants, highlighting the significance of these processes in the context of food safety and public health (13–16).

Transposon sequencing (Tn-seq) approaches have been used to determine the mechanisms through which bacteria survive in different environments. Tn-seq was used to identify the genes involved in *Pseudomonas simiae* colonization of plant roots, which highlighted the importance of genes involved in flagella production, cell envelope biosynthesis, carbohydrate metabolism, and amino acid transport and metabolism (17). A similar Tn-Seq approach was used to determine the genes required for *Salmonella* colonization of tomatoes, identifying a high abundance of mutants associated with amino acid biosynthesis (18). We have previously used another transposon sequencing approach, TraDIS-*Xpress*, to find the genes involved in biofilm formation in *Escherichia coli* (19) and *S. enterica* serovar Typhimurium (20) on glass over time. TraDIS-*Xpress* builds on conventional transposon sequencing approaches by using larger denser transposon mutant libraries and by incorporating an outwards-transcribing promoter into the transposon element (21). Induction of this promoter enables increased the expression of genes downstream of transposon insertions, thereby facilitating investigation into how the expression, as well as gene disruption, affects the survival of the mutant in a given condition. This approach also allows for the analysis of essential genes that do not tolerate insertional inactivation by transposons and can, therefore, not be assayed with conventional tools.

In this study, we established an alfalfa plant colonization model that was used in conjunction with TraDIS-*Xpress* to investigate gene essentiality in *Salmonella* establishment on alfalfa over time. A library of *S*. Typhimurium transposon mutants was cultivated on sprouted alfalfa plants, and cells were isolated at different stages to identify the genes involved in the establishment of plant development *in planta* over time. Comparisons were made with findings from our previous study focusing on biofilm formation on glass surfaces (20). This allowed for the identification of plant-specific and glass-specific mechanisms used by *S*. Typhimurium to establish in biotic and abiotic surfaces, as well as conserved genes that play crucial roles on both surfaces.

We showed variations in the importance of factors, including flagella biosynthesis, LPS production, and stringent response regulation in establishment on plants versus glass surfaces. Understanding the genes involved in the colonization of both biotic and abiotic surfaces over time provides valuable insights for the development of targeted antibacterial therapeutics to enhance food safety throughout the food processing chain.

## RESULTS

### Establishment of an alfalfa plant colonization model

**Fig 1:**
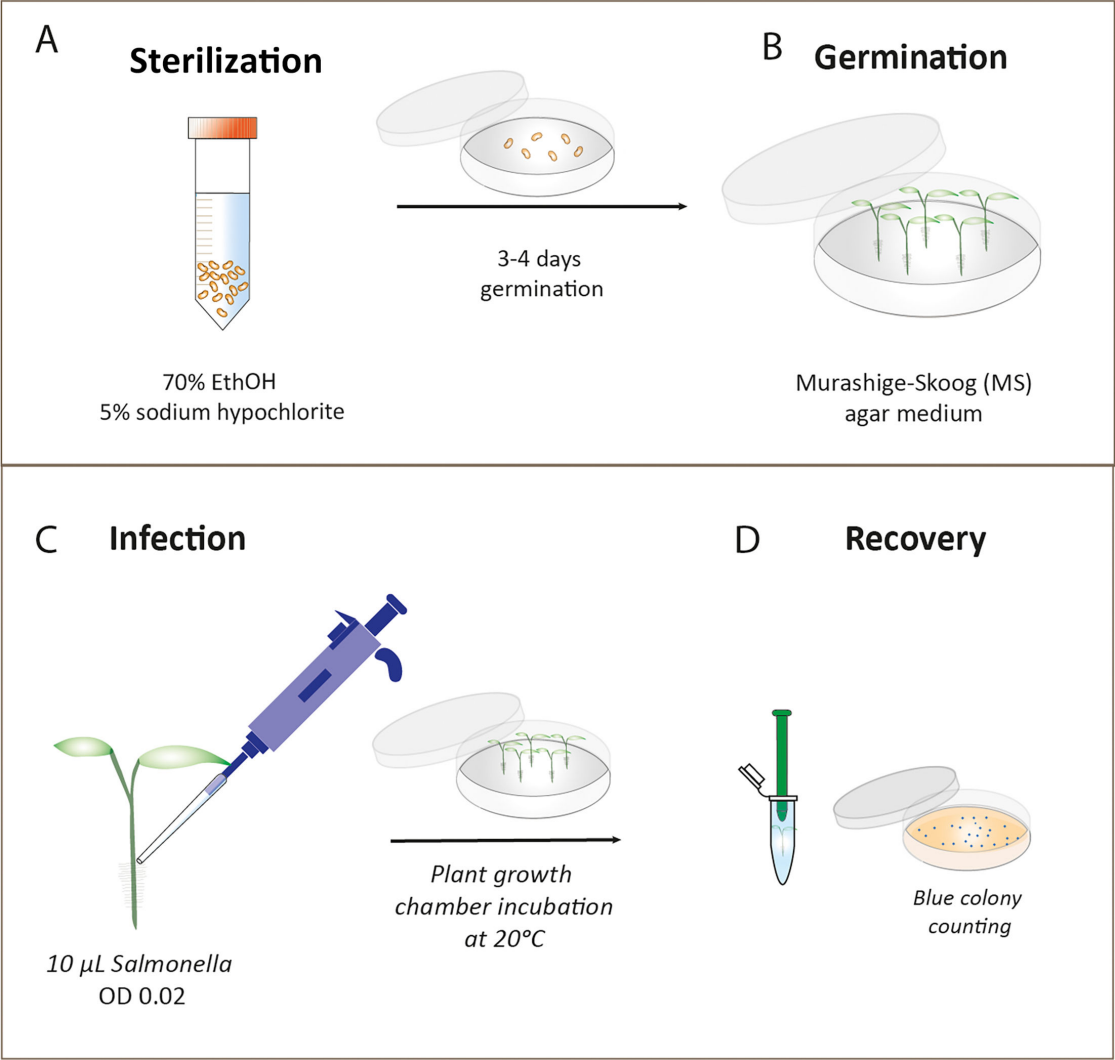
Alfalfa plant colonization model. (A) Alfalfa seeds were sterilized by immersion in 70% ethanol for 30 s, followed by washing in 5% sodium hypochlorite for 3 min. (B) Subsequently, the sterilized seeds were left to germinate in darkness at 20°C in Murashige–Skoog (MS) agar medium for 3–4 days. (C) The seedlings were inoculated at the root–shoot intersection using 10 μL of *Salmonella* inoculum, normalized to an optical density (OD) of 0.02. Inoculated seedlings were then transferred to fresh MS plates and incubated in a benchtop plant growth chamber at 20°C. (D) To facilitate selection via blue colony screening, *Salmonella* recovery and quantification were performed over time using the 14028*S::lacIZ* strain. Inoculated seedlings were homogenized by mechanical disruption using a pestle to release the bacterial cells. Cell suspensions were subjected to serial dilution and plated onto X-gal/IPTG LB plates for further analysis.

To assess the ability of *S*. Typhimurium to establish and proliferate on plant hosts, an alfalfa seedling model was established (Fig. 1). Initially, seeds underwent sterilization and were allowed to germinate in Murashige–Skoog (MS) medium for 3 days (Fig. 1A and B). Following this germination period, the seedlings were inoculated at the root–shoot intersection with a *S*. Typhimurium strain marked with the *lacZ* reporter gene (14028*S::lacIZ*) for blue colony selection and counting (Fig. 1C and D).

### *Salmonella* effectively colonizes alfalfa sprouts and increases in numbers over time

**Fig 2:**
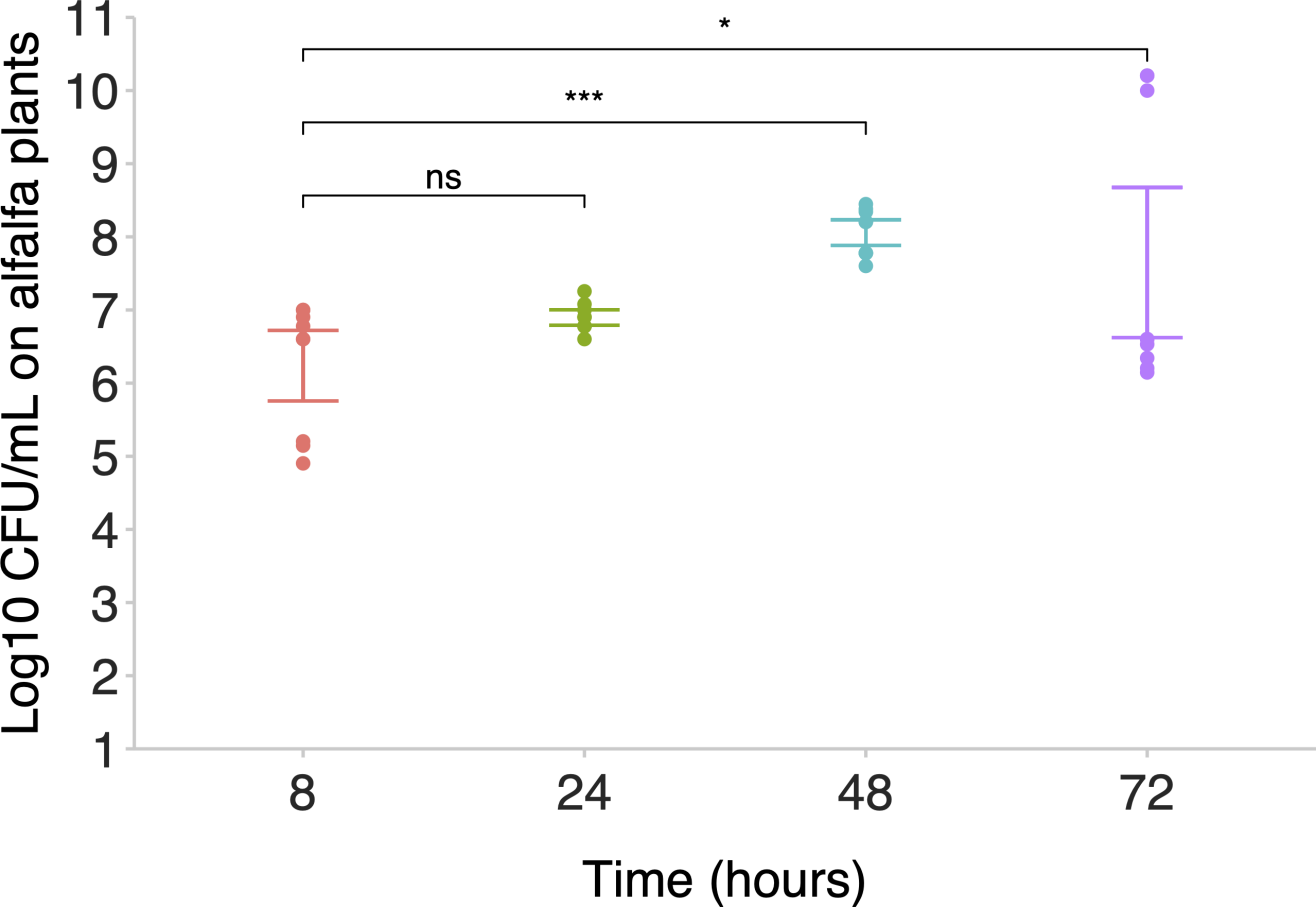
*Salmonella* effectively colonizes the alfalfa model. *S*. Typhimurium was isolated from alfalfa seedlings following 8-, 24-, 48- and 72-h post-inoculation, and CFU/mL was determined at each time point. Points represent three biological and three technical replicates, and error bars show 95% confidence intervals. Asterisks show a significant difference (Student’s *t*-test) in CFU/mL from the 8-h time point: ns not significant, * *P* < 0.05, ** *P* < 0.01, *** *P* < 0.001, **** *P* < 0.0001.

To investigate the effectiveness of *Salmonella* colonization in alfalfa seedlings, a strain tagged with *lacZ* (14028*S::lacIZ*) (22) was used to inoculate seedlings 3 days after germination. Following inoculation, the seedlings were homogenized, and CFU/mL per seedling was quantified. The cells were recovered after growth for 8, 24, 48, and 72 h, demonstrating a significant increase in *S*. Typhimurium colonization of alfalfa over time (see Fig. 2).

### Genes involved in *Salmonella* establishment on alfalfa over time

**Fig 3:**
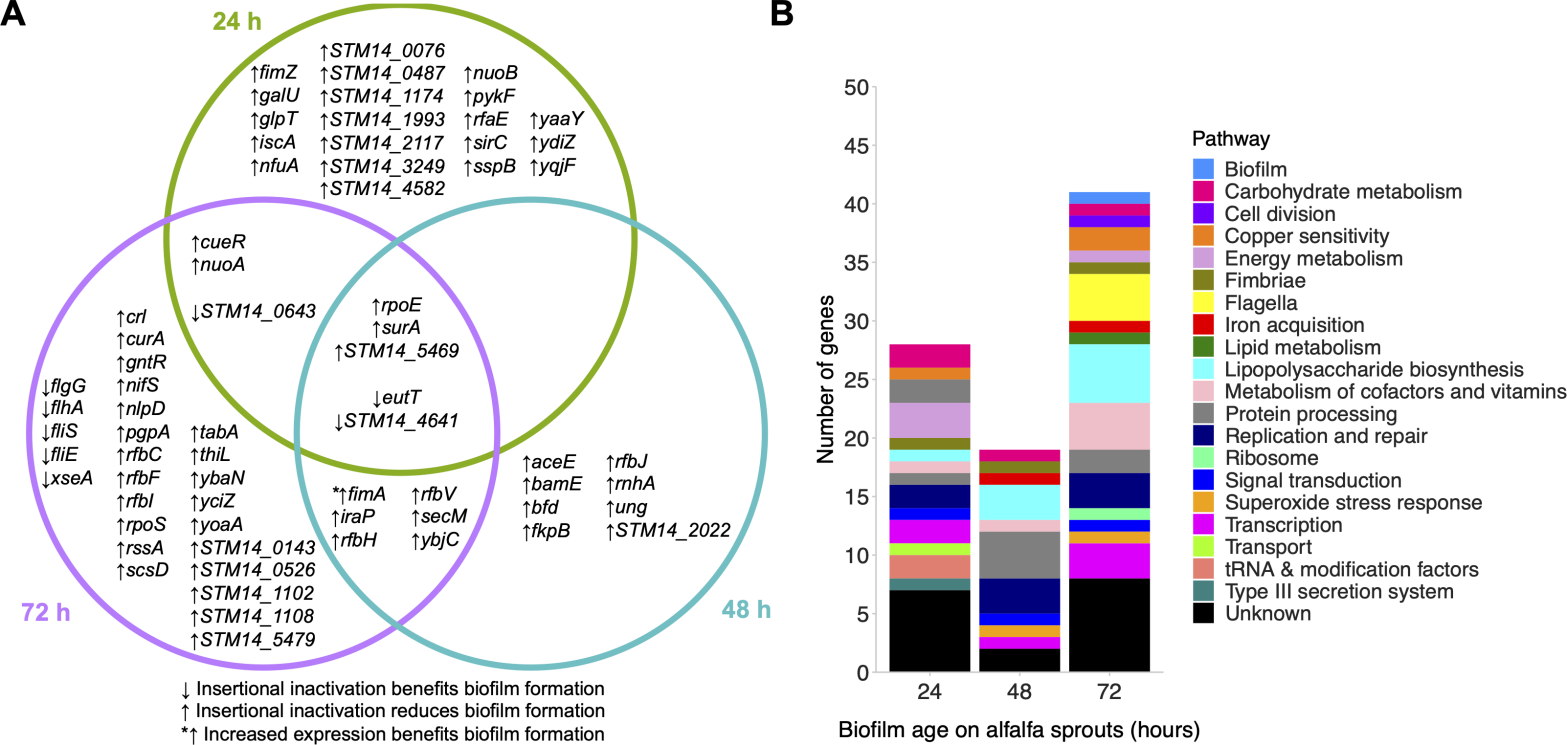
(A) Genes and (B) pathways identified by TraDIS-*Xpress* to be involved in alfalfa colonization 24-, 48-, and 72-h post-inoculation.

TraDIS-*Xpress* was used to identify genes involved in alfalfa colonization by *S*. Typhimurium for 3 days (24-, 48- and 72-h post-seeding). These timepoints were carefully considered to capture the potentially diverse mechanisms required by *Salmonella* at different stages of alfalfa colonization. This includes the early stages involving initial attachment and microcolony formation (at 24 h) and the subsequent phases of *Salmonella* establishment on alfalfa (spanning 48–72 h). We identified 69 genes involved in *S*. Typhimurium colonization and establishment on alfalfa sprouts over time (Table S1). These included genes involved in LPS biosynthesis, DNA housekeeping, respiration, and stress response (Fig. 3). Variation in insertion frequency per gene between replicates was low, indicating low experimental error (Fig. S1).

Genes involved in adhesion were identified as beneficial after 24-h growth, including previously reported genes, such as a negative fimbrial regulator *fimZ* (23) and type III secretion system component *sirC* (24). After 48 h, genes involved in DNA housekeeping (*rnhA* and *ung*) (25, 26), iron storage (*bfd*) (27), and outer membrane protein assembly (*bamE*) (28) benefit the further establishment of *Salmonella* on alfalfa. After 72 h, genes associated with roles in LPS O-antigen production (*rfbF, rfbI, rfbC, rfbV*, and *rfbH*) (29), flagella biosynthesis (*flgG, flhA, fliS*, and *fliE*) (30), and stress response (*rpoS, iraP*, and *crl*) (31) were identified.

Five genes were shared among the time points tested: *eutT, surA, rpoE*, *STM14_4641*, and *STM14_5469*. Preventing the function of the *eut* operon through disruption of *eutT* (32) was beneficial to *S*. Typhimurium establishment at all time points tested. Transcription of *STM14_4641* encoding an RNA-directed DNA polymerase was detrimental to colonization throughout its growth on alfalfa sprouts. Transposon mutants were fewer across all time points in *surA* (outer membrane protein chaperone) (33), *rpoE* (sigma factor involved in response to misfolded protein stress) (34), and *STM14_5469* (unknown function) relative to planktonic controls, suggesting that these genes are beneficial throughout all stages of alfalfa colonization.

### Conserved pathways crucial for *Salmonella* establishment on alfalfa sprouts and glass

**Fig 4:**
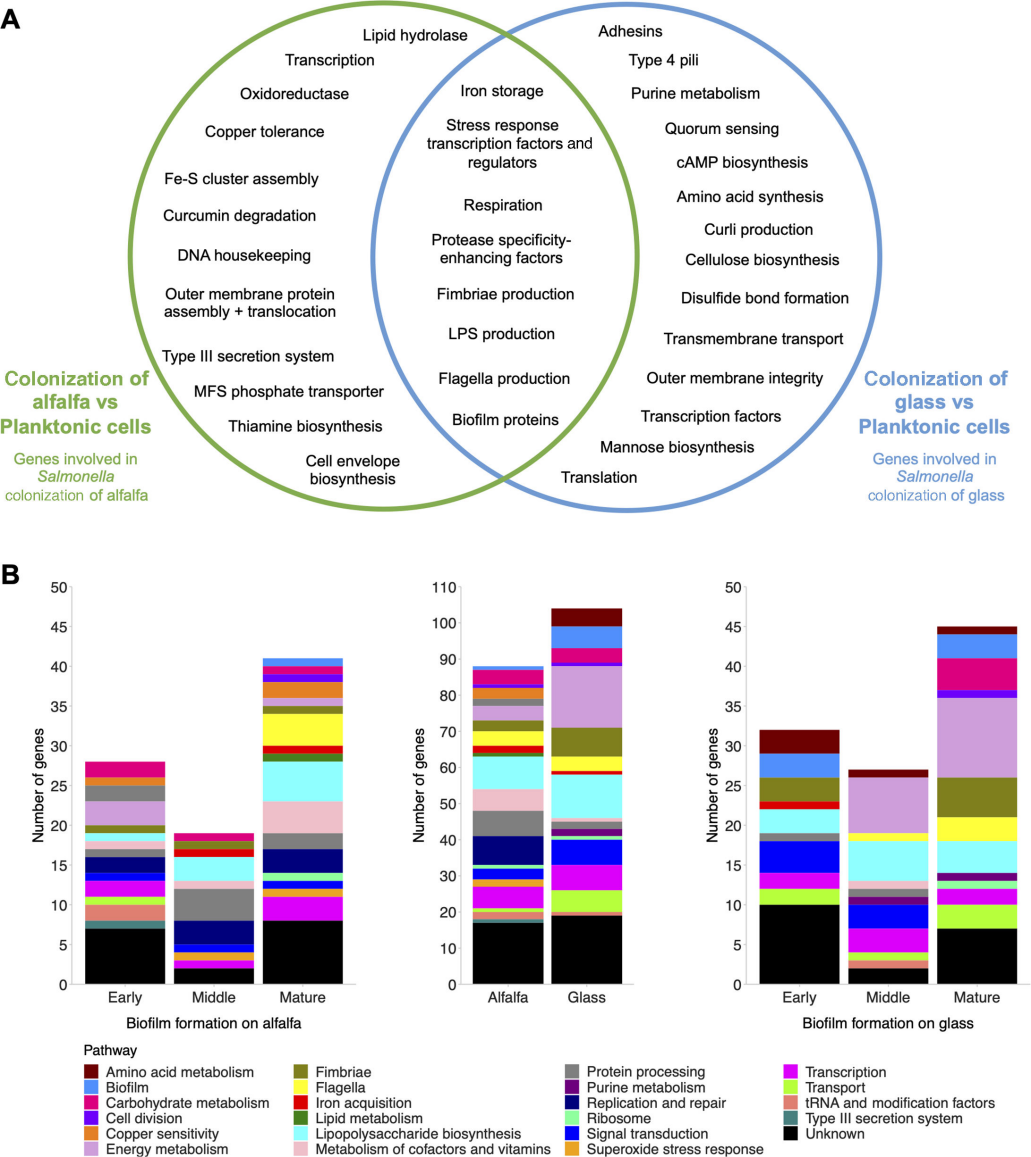
(A) Conserved and surface-specific pathways involved in *S.* Typhimurium colonization of alfalfa sprouts and glass. (B) Abundance of genes in each pathway for *Salmonella* grown on alfalfa sprouts or glass over time.

We have previously identified genes essential for biofilm formation on glass over time using the same *S*. Typhimurium transposon mutant library used in this study (20). The library used has 500,000 unique insertion sites, corresponding to approximately one insertion every eight base pairs. Insertion frequencies in mutant libraries colonizing glass or plant surfaces were both compared to planktonic cultures grown for the same amount of time. This acted as a standard to demonstrate where transposon insertions affected surface colonization relative to planktonic growth, and the subsequent gene lists for bacterial communities at the same developmental stages on each surface were then compared. These found pathways involved in *S*. Typhimurium establishment on both surfaces included flagella biosynthesis, LPS production, respiration, iron storage, and stress response. Seven genes were conserved between growth on alfalfa sprouts and on glass (Fig. 4). These were *nuoA* and *nuoB*, involved in the synthesis of the first NADH hydrogenase in the electron transport chain (35), fimbrial subunit *fimA* and its regulator *fimZ* (23), *rfbJ* involved in LPS O-antigen synthesis (29), *ybaN* predicted to have a role in iron acquisition (36), and stress response sigma factor *rpoS* (37). The ethanolamine utilization pathway played an important role in *S*. Typhimurium establishment on both alfalfa sprouts (*eutT*) and on glass (*eutQ*) at all time points tested, with disruption of each gene aiding colonization. Together, this reveals a core set of pathways involved in the colonization of both biotic and abiotic surfaces (Fig. 4).

### Differential flagella and lipopolysaccharide biosynthesis in alfalfa vs. glass

Deletion mutants were constructed in targets identified by TraDIS-*Xpress* to investigate their effects on colonization and establishment on the two surfaces (biotic and abiotic). These mutants were subjected to competitive colonization experiments with wild-type *S*. Typhimurium strains on both glass and alfalfa surfaces. Equal numbers of mutant and wild-type CFU/mL were inoculated onto glass beads and alfalfa plant sprouts. Subsequently, the percentage change in mutant CFU within the recovered populations from each surface was determined over time.

**Fig 5:**
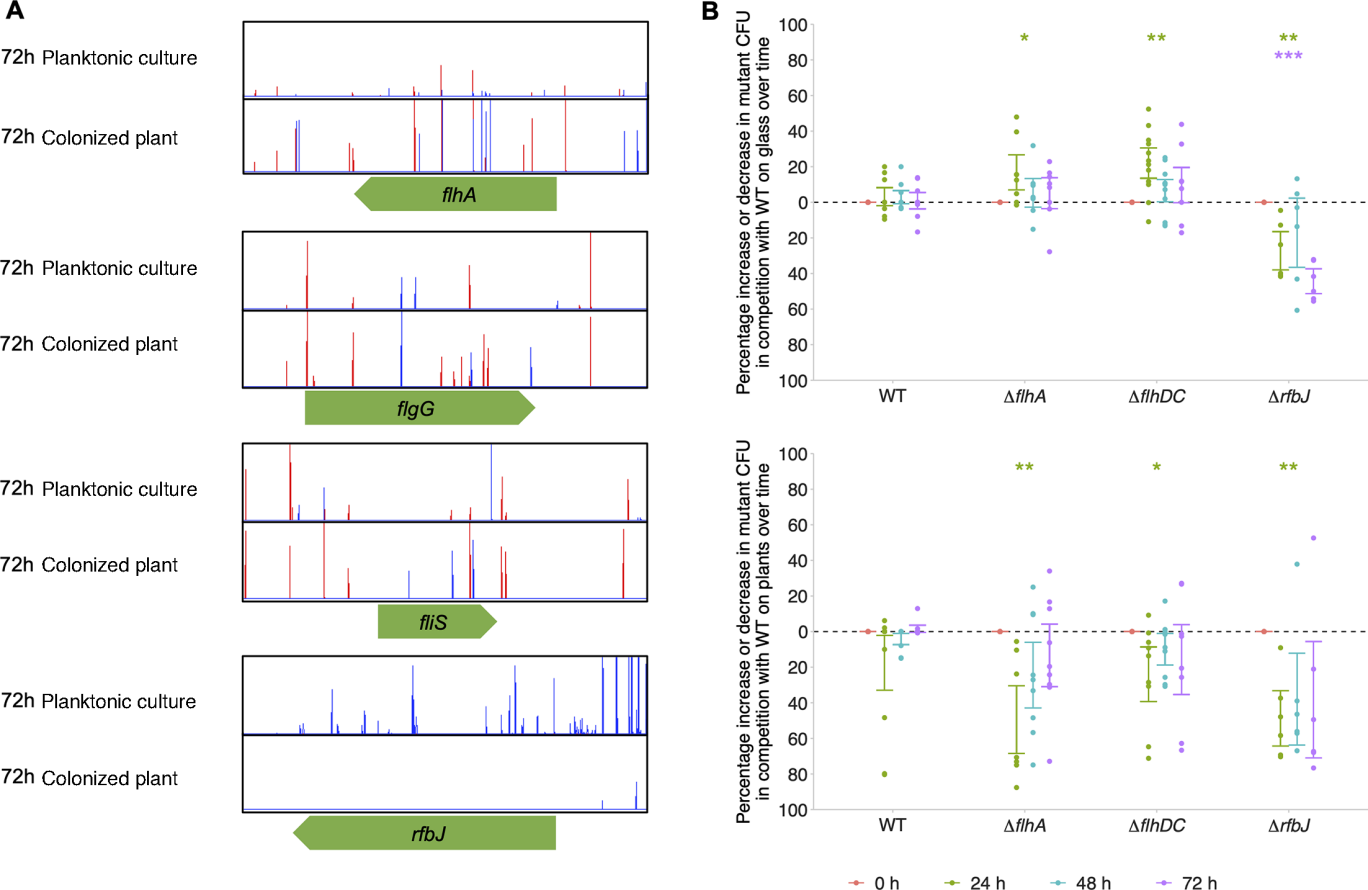
(A) Insertion loci and frequency in and around genes involved in flagella biosynthesis (*flhA*, *flgG*, and *fliS*) and LPS O-antigen biosynthesis (*rfbJ*) following growth on alfalfa sprouts relative to planktonic growth. Red lines indicate that the transposon-located promoter is facing left-to-right, and blue lines indicate that it is oriented right-to-left. The images are representative of two independent replicates. (B) Percentage increase or decrease in *flhA*, *flhDC*, and *rfbJ* deletion mutants in biofilms formed on glass (top panel) and alfalfa plant sprouts (bottom panel) in competition with wild-type (WT) *S.* Typhimurium. Points show changes in the percentage of mutant CFU relative to time point 0 and show three technical and four biological replicates. Error bars denote 95% confidence intervals, and asterisks show significant differences (one-sample *t*-test, change from 0) of each mutant from time point 0, where time points are distinguished by color: * *P* < 0.05, ** *P* < 0.01, *** *P* < 0.001, ****P < 0.0001.

TraDIS-*Xpress* indicated that inactivation of genes involved in flagella biosynthesis was beneficial for plant colonization after 72 h of growth (Fig. 5A). We predicted that because flagella are detected by the plant’s immune system, aflagellated cells will have a competitive advantage in these communities during colonization. Our previous work suggested that aflagellated cells were disadvantaged at colonizing glass surfaces (20). To characterize the role of flagella in *S*. Typhimurium establishment in both environments, a deletion mutant of the main flagella biosynthetic regulator (*flhDC*) and a component of the flagella export machinery (*flhA*) were grown on glass and alfalfa sprouts in competition with wild-type *S*. Typhimurium. At the initial stages of colonization (24-h post-inocculation), Δ*flhDC* and Δ*flhA* exhibited a significantly enhanced competitive advantage at the colonizing glass but were competitively disadvantaged at colonizing alfalfa plants (Fig. 5B), contrary to the TraDIS-*Xpress* findings.

LPS core and O-antigen biosynthesis genes were beneficial for growth on alfalfa sprouts; however, the impact of different LPS biosynthesis genes on *S*. Typhimurium colonization varied. Some exhibited beneficial effects when inactivated during glass colonization, whereas others had detrimental impacts. Based on the TraDIS-*Xpress* data, *rfbJ* was beneficial for growth and establishment on alfalfa sprouts, whereas gene inactivation was beneficial for establishment on glass. We created a deletion mutant of *rfbJ* in *S*. Typhimurium to investigate its effect on glass and plant colonization. Deletion of *rfbJ* resulted in reduced colonization of both glass and plant over time (Fig. 5B). This indicates the importance of this gene for adhesion and colonization of both surfaces.

### Genes involved in copper tolerance, type III secretion regulation, and curcumin degradation conferred a competitive advantage to *Salmonella* establishment on alfalfa

Analysis of the TraDIS-*Xpress* data found pathways involved in *S*. Typhimurium establishment on alfalfa plants that were not involved during biofilm formation on glass. These included type III secretion regulation (*sirC*) (24) and Fe–S cluster assembly (*iscA*) (38), which were beneficial at the early stages of colonization of alfalfa. Curcumin degradation (*curA*) (39) was beneficial following 72-h growth on alfalfa, and copper tolerance (*cueR*) (40) was beneficial following 24- and 72-h growth on alfalfa.

**Fig 6:**
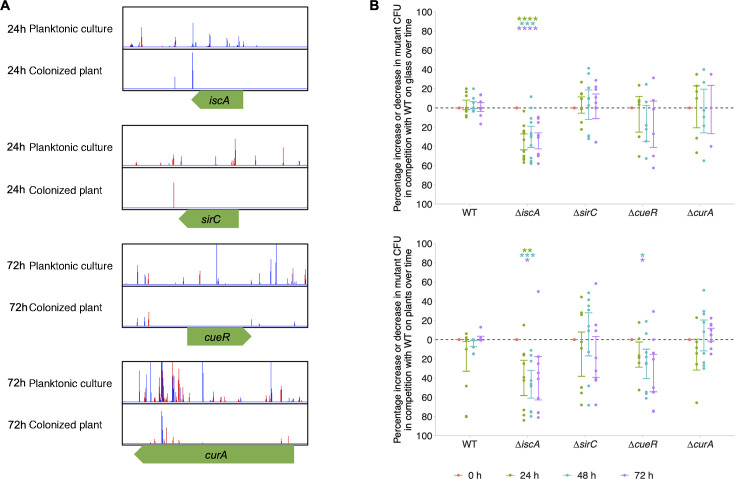
(A) Transposon insertions within and around *iscA*, *sirC*, *cueR* , and *curA* in *S.* Typhimurium planktonic culture compared with *Salmonella* recovered from alfalfa after 24 or 72 h. Lines show the insertion loci, and the height of the lines shows the number of reads mapped to the loci. The color of the line indicates the orientation of the promoter within the transposon: red lines denote the promoter is promoting transcription left-to-right, and blue lines denote right-to-left. Plot files shown are representative of two independent replicates. (B) Percentage increase or decrease in *iscA*, *sirC*, and *cueR* deletion mutants in biofilms formed on glass (top panel) and alfalfa plant sprouts (bottom panel) in competition with wild-type (WT) *S.* Typhimurium. Points show changes in the percentage of mutant CFU relative to time point 0 and show three technical and four biological replicates. Error bars denote 95% confidence intervals, and asterisks show significant differences (one-sample *t*-test, change from 0) of each mutant from time point 0, where time points are distinguished by color: * *P* < 0.05, ** *P* < 0.01, *** *P* < 0.001, **** *P* < 0.0001.

Gene deletion mutants were made in these genes and grown in the presence of wild-type *S*. Typhimurium on glass and alfalfa plants to investigate their effects on colonization. Deletion of *iscA* resulted in a competitive disadvantage for colonization of both glass and alfalfa plants, supporting the TraDIS-*Xpress* findings (Fig. 6B). Deletion of *cueR* caused a competitive disadvantage in the colonization of alfalfa plants, but no significant change was observed in glass colonization, demonstrating that *cueR* expression is only beneficial for colonization of plant surfaces and not glass surfaces. No significant change was observed in the percentage of Δ*sirC* or Δ*curA* mutants over time on either glass or plants, suggesting that the effects of these genes on colonization observed in the TraDIS-*Xpress* data cannot be quantified by this assay.

## DISCUSSION

The primary objective of this study was to identify the mechanisms utilized by *S*. Typhimurium to colonize effectively and establish fresh produce and compare these to the pathways required for colonization and biofilm formation on glass, across various stages of colonization. To achieve this, we established a fresh-produce alfalfa colonization model and used genome-wide transposon insertion sequencing (TraDIS-*Xpress*) to investigate *S*. Typhimurium establishment on alfalfa, comparing our findings to mechanisms previously identified for biofilm formation on glass surfaces (20). Our aim was to discern the extent to which these mechanisms are universally necessary for adhesion, colonization, and establishment on biotic surfaces in contrast to abiotic surfaces. Our working hypothesis centered on the presence of both common and distinct mechanisms in the two tested environments. Several key findings emerge from this study.

We found that differences in gene essentiality differed over time as *S*. Typhimurium colonized the alfalfa in a similar way to which was seen on glass surfaces. Initially, we identified the importance of genes involved in adhesion and type III secretion systems, and over time, genes involved in DNA housekeeping and envelope synthesis became more important for establishment. In the latest colonization timepoint tested, genes involved in LPS synthesis, flagella synthesis, and global stress response systems were key to *S*. Typhimurium establishment on alfalfa. We identified seven conserved genes important in *S*. Typhimurium establishment on both alfalfa sprouts and glass, highlighting the shared genetic elements critical for *S*. Typhimurium colonization of diverse surfaces. These genes belong to various functional categories, including NADH hydrogenase synthesis (*nuoA* and *nuoB*), fimbrial regulation and production (*fimA* and *fimZ*), LPS O-antigen synthesis (*rfbJ*), iron acquisition (*ybaN*), and stress responses (*rpoS*). Ethanolamine utilization genes, *eutT* and *eutQ*, were also identified to play an important role in *S*. Typhimurium establishment on both environments, with their disruption aiding colonization of both surfaces. Notably, ethanolamine signaling has been reported to aid *S*. Typhimurium infection of mammalian cells (41). The identification of these conserved genes underscores their significance in surface colonization, regardless of the surface material.

Flagella biosynthesis affects the colonization of biotic and abiotic surfaces differently in our study. We showed that aflagellated mutants (*ΔflhDC* and *ΔflhA*) exhibit significantly enhanced glass colonization at the early stages of colonization (24 h) but perform significantly worse on alfalfa. However, with time, these mutants regain their ability to grow on alfalfa. This demonstrates the potential role of the flagellum in the initial stages of adhesion to alfalfa. We know that flagellar motility is essential for initial host colonization in several bacterial species (42, 43). This contrasts with TraDIS-*Xpress* results, highlighting the complexity of the role of flagella at different stages of colonization and the adaptive capabilities of *S*. Typhimurium over time (44).

We also found pathways involved in *S*. Typhimurium establishment on alfalfa seedlings that were not involved in biofilm formation on glass. Notably, genes related to type III secretion regulation (*sirC*), Fe–S cluster assembly (*iscA*), curcumin degradation (*curA*), and copper tolerance (*cueR*) confer a competitive advantage to *S*. Typhimurium during colonization of alfalfa. Deletion of *cueR* reduced the ability of *S*. Typhimurium to colonize plants but had no effect on glass, demonstrating conditional importance between surfaces. Metals play an important role in plant–pathogen interactions (45), and regulating the expression of copper export through *cueR* is therefore beneficial for colonization and establishment on a plant. Deletion of *iscA* reduced colonization on both glass and plant surfaces, and colonization was not different in Δ*sirC* or Δ*curA* deletion mutants. TraDIS-*Xpress* can determine very small changes in competitive fitness that may not always be seen in culture-based assays; therefore, further characterization is needed to determine how these genes affect plant colonization.

The use of mixed pools of mutants in TraDIS-*Xpress* experiments offers several advantages, primarily by better simulating the complexity of environmental communities composed of multiple strains and species. This approach is more representative of real-world populations compared with isogenic populations typically studied *in vitro*. However, this comes with limitations, particularly for follow-up target characterization. Differences between polygenic and isogenic populations can result in discrepancies when comparing data from whole gene deletion mutants and TraDIS-*Xpress* data. Microbes form complex communities and structures (such as biofilms) that can be influenced by various factors affecting their fitness over time. Consequently, differences between gene deletion mutants and the wild type may not always be readily detectable in simple culture-based assays.

In conclusion, this research provides a comprehensive understanding of the genetic determinants that influence *S*. Typhimurium colonization and establishment on diverse surfaces. The findings emphasize the role of specific genes at different stages of *S*. Typhimurium colonization of fresh produce, reflecting its adaptability and the conditional importance of certain pathways. Moreover, the identification of conserved genes highlights their significance in the pathogen’s establishment on various substrates. This knowledge is invaluable in advancing our understanding of *Salmonella* pathogenesis and host–microbe interactions and may have implications for controlling *Salmonella* colonization and infection.

## MATERIALS AND METHODS

### Alfalfa seed sterilization and germination

Alfalfa seeds were sterilized by immersion in 20 mL of 70% ethanol for 30 s, followed by three sequential rinses with 20-mL sterile water. Subsequently, the seeds were treated with 5% sodium hypochlorite (20 mL) for 3 min on a rolling platform. Three subsequent washes in water were carried out. For germination, sterilized seeds were transferred to square agar plates (20 mL) containing Murashige–Skoog (MS) agar medium. These seeds were positioned with sufficient spacing to allow for 3 days of germination, reaching an approximate size of 1 cm. Following germination, the seedlings were transferred to fresh MS plates and inoculated with *S*. Typhimurium. Adequate seedlings were included in the process to enable replication for experimental purposes.

### Quantification of *Salmonella* on alfalfa seedlings

Three-day-old alfalfa seedlings were inoculated with 10-µL *S. enterica* subsp. *enterica* serovar Typhimurium strain 14028*S* tagged with the *lacZ* operon (14028*S::lacIZ*) (22), with the bacterial density normalized to an optical density (OD_600nm_) of 0.02. The seedlings were incubated at 20°C throughout the experiment. After 8-, 24-, 48-, and 72-h post-inoculation, three seedlings per timepoint were homogenized using a plastic pestle in PBS and then serially diluted in PBS. The dilutions were spotted on LB-agar plates supplemented with 40 µg/mL X-gal (-bromo-4-chloro-3-indolyl β-D-galactopyranoside) and 1 mM IPTG (isopropyl β-D-1-thiogalactopyranoside), which allows *S.* Typhimurium tagged with *lacZ* to appear blue. The prepared plates were incubated at 37°C overnight. Following overnight incubation, colony-forming units (CFU) were counted. Each time point included at least three technical replicates and three biological samples, ensuring robust and reliable quantification of *S*. Typhimurium populations.

### Competition assays on alfalfa seedlings and glass

Single gene deletion mutants were made following the gene doctoring protocol (46) using plasmids constructed via Golden Gate assembly (47). The mutants were validated by whole genome sequencing on NextSeq2000 (Illumina), aiming for a 60× coverage to confirm the loss of the gene of interest. Sequencing files were assembled into contigs using Shovill (version 1.1.0) (48) and mapped against a reference genome (CP001363) to validate the loss of the target gene. Primers for mutant construction are listed in Table S2. For competition in alfalfa seedlings, 3-day-old seedlings were inoculated with 10 µL of *S*. Typhimurium tagged with *lacZ* (14028*S::lacZ*) in a 1:1 ratio with deletion mutants, all adjusted to a final OD of 0.02 in 10 mM MgCl_2_. Inoculated seedlings were subsequently transferred to fresh MS plates and incubated at 20°C. After 24-, 48-, and 72-h post-inoculation, three seedlings per timepoint were homogenized using a plastic pestle in PBS and then serially diluted in PBS. The dilutions were spotted on LB-agar plates supplemented with 40 µg/mL X-gal and 1 mM IPTG. For competition on glass beads, beads suspended in 5 mL of LB-NaCl were inoculated with 50 µL of selected strains mixed with 14028*S::lacZ* in a 1:1 ratio, normalized to a final OD of 0.02. After incubation, three beads were recovered at 24-, 48-, and 72-h post-inoculation, washed in PBS to eliminate planktonic growth, and the biofilm cells were recovered by vortexing in PBS. The recovered cells were serially diluted and spotted on LB-agar plates supplemented with 40 µg/mL X-gal and 1 mM IPTG.

### TraDIS-*Xpress* library preparation, sequencing, and data analysis

Three-day-old alfalfa seedlings, grown on MS agar, were inoculated at the shoot–root junction with a 10-µL droplet of a *S*. Typhimurium transposon mutant library (described by Holden, Yasir) (20), normalized to an OD_600nm_ of 0.01 with 1 mM IPTG to induce transcription from the transposon-located promoter. Seedlings were then allowed to grow at 30°C (for the results to be directly comparable to growth on glass beads) before sampling following 24-, 48- and 72-h growth. Ten seedlings were processed per timepoint and were homogenized in 1 mL of sterile PBS using a plastic pestle. Samples were filtered through 5-µm syringe filters to isolate bacterial cells and eliminate plant cell contamination. Genomic DNA was extracted from these cells following the protocol described by Trampari, Holden (49). A Mu sSeek DNA fragment library preparation kit (ThermoFisher) was used to tagment genomic DNA and then purified with AMPure XP beads (Beckman Coulter). DNA fragments were amplified using customized primers that anneal to the tagmented ends and biotinylated primers that anneal to the transposon. These PCR products were purified, and biotinylated DNA was incubated for 4 h with streptavidin beads (Dynabeads kilobaseBINDER, Invitrogen) to capture only DNA fragments containing the transposon. These fragments were amplified using barcoded sequencing primers that anneal to the tagmented ends and transposon (21). DNA fragments were then purified and size-selected using AMPure beads. Fragment length was quantified using a Tapestation (Aligent) and sequenced on a NextSeq500 using the NextSeq 500/550 High Output Kit v2.5 with 75 cycles. Fastq files were aligned to the *S*. Typhimurium 14028*S* reference genome (CP001363, modified to include chromosomally integrated *lacIZ*) using BioTraDIS (version 1.4.3) (50). Significant differences (*P* < 0.05, after correction for false discovery) in insertion frequencies between planktonic and *Salmonella* recovered from glass and alfalfa at each time point were found using BioTraDIS and AlbaTraDIS (version 1.0.1) (51). Amino acid sequences for genes of unknown function were analyzed using EggNOG (version 5.0.0) (52) to determine the predicted function.

## Data Availability

Nucleotide sequence data supporting the analysis in this study have been deposited in ArrayExpress under the accession numbers E-MTAB-13495 (colonization of alfalfa plants) and EMTAB-11765 (colonization of glass beads). The authors confirm all supporting data, code, and protocols have been provided within the article or through supplemental data files.

## SUPPLEMENTAL MATERIAL

Supplemental material aem.00139-24-s0001.docx Tables S1 and S2; Figure S1.

ASM does not own the copyrights to Supplemental Material that may be linked to, or accessed through, an article. The authors have granted ASM a non-exclusive, world-wide license to publish the Supplemental Material files. Please contact the corresponding author directly for reuse.
